# Supportive Care During Pediatric Hematopoietic Stem Cell Transplantation: Prevention of Infections. A Report From Workshops on Supportive Care of the Paediatric Diseases Working Party (PDWP) of the European Society for Blood and Marrow Transplantation (EBMT)

**DOI:** 10.3389/fped.2021.705179

**Published:** 2021-07-29

**Authors:** Marianne Ifversen, Roland Meisel, Petr Sedlacek, Krzysztof Kalwak, Luisa Sisinni, Daphna Hutt, Thomas Lehrnbecher, Adriana Balduzzi, Tamara Diesch, Andrea Jarisch, Tayfun Güngör, Jerry Stein, Isaac Yaniv, Halvard Bonig, Michaela Kuhlen, Marc Ansari, Tiago Nava, Jean-Hugues Dalle, Cristina Diaz-de-Heredia, Eugenia Trigoso, Ulrike Falkenberg, Mihaela Hartmann, Marco Deiana, Marta Canesi, Chiara Broggi, Alice Bertaina, Brenda Gibson, Gergely Krivan, Kim Vettenranta, Toni Matic, Jochen Buechner, Anita Lawitschka, Christina Peters, Akif Yesilipek, Koray Yalçin, Giovanna Lucchini, Shahrzad Bakhtiar, Dominik Turkiewicz, Riitta Niinimäki, Jacek Wachowiak, Simone Cesaro, Arnaud Dalissier, Selim Corbacioglu, Andre Manfred Willasch, Peter Bader

**Affiliations:** ^1^Department of Paediatrics and Adolescent Medicine, Copenhagen University Hospital Rigshospitalet, Copenhagen, Denmark; ^2^Division of Pediatric Stem Cell Therapy, Department of Pediatric Oncology, Hematology and Clinical Immunology, Medical Faculty, Heinrich-Heine-University, Duesseldorf, Germany; ^3^Department of Pediatric Hematology and Oncology, Hospital Motol, Charles University, Prague, Czechia; ^4^Department of Pediatric Hematology, Oncology and Bone Marrow Transplantation, Wroclaw Medical University, Wroclaw, Poland; ^5^Pediatric Hematology, Oncology and Hematopoietic Stem Cell Transplantation Unit, Hospital Santa Creu i Sant Pau, Barcelona, Spain; ^6^Division of Pediatric Hematology, Oncology and Bone Marrow Transplantation, The Edmond and Lily Safra Children's Hospital, Tel Aviv, Israel; ^7^Division for Pediatric Hematology and Oncology, Hospital for Children and Adolescents, University Hospital, Goethe University, Frankfurt am Main, Germany; ^8^Clinica Pediatrica Università degli Studi di Milano Bicocca, Fondazione Monza e Brianza per il Bambino e la sua Mamma, Ospedale San Gerardo, Monza, Italy; ^9^Division of Pediatric Hematology-Oncology, University Children's Hospital of Basel, Basel, Switzerland; ^10^Division for Stem Cell Transplantation, Immunology and Intensive Care Medicine, Hospital for Children and Adolescents, University Hospital, Goethe University, Frankfurt am Main, Germany; ^11^Department of Hematology, Immunology, Oncology and Stem Cell Transplantation, University Children's Hospital Zürich, Zurich, Switzerland; ^12^Division of Pediatric Hematoloy-Oncology, Schneider Children's Medical Center of Israel, Petach Tikva, Israel; ^13^Institute for Transfusion Medicine and Immunohematology of Goethe University, German Red Cross Blood Service Baden-Württemberg-Hessen, Frankfurt am Main, Germany; ^14^Paediatrics and Adolescent Medicine, Medical Faculty, University of Augsburg, Augsburg, Germany; ^15^Division of Pediatric Hematology-Oncology, University Hospital of Geneva, Geneva, Switzerland; ^16^Cansearch Research Platform in Paediatric Oncology and Haematology, Department of Paediatrics, Gynaecology and Obstetrics, Faculty of Medicine, University of Geneva, Geneva, Switzerland; ^17^Hematology and Immunology Department, Robert-Debre Hospital, Assistance Publique-Hopitaux de Paris and University of Paris, Paris, France; ^18^Department of Pediatric Oncology and Hematology, Hospital Universitari Vall d'Hebron, Barcelona, Spain; ^19^Paediatric Transplant Unit, Hospital University and Polytechnic, Hospital LA FE, Valencia, Spain; ^20^Stem Cell Transplantation-Unit, Department of Pediatrics, St. Anna Children's Hospital, Medical University of Vienna, Vienna, Austria; ^21^Paediatric Haematology-Oncology Department, Istituto di Ricovero e Cura a Carattere Scientifico G Gaslini, Genova, Italy; ^22^Department of Pediatric Hematology-Oncology and Cell and Gene Therapy, Istituto di Ricovero e Cura a Carattere Scientifico, Ospedale Bambino Gesù, Rome, Italy; ^23^Division of Stem Cell Transplantation and Regenerative Medicine, Department of Pediatrics, School of Medicine, Stanford University, Stanford, CA, United States; ^24^Department of Paediatric Haematology–Oncology, Royal Hospital for Children, Glasgow, United Kingdom; ^25^Central Hospital of Southern Pest, National Institute of Hematology and Infectious Disease, Budapest, Hungary; ^26^Children's Hospital and Pediatric Research Center, University of Helsinki, Helsinki, Finland; ^27^Department of Pediatrics, University Hospital Center Zagreb, Zagreb, Croatia; ^28^Department of Pediatric Hematology and Oncology, Oslo University Hospital, Oslo, Norway; ^29^Department of Pediatric Hematology and Pediatric Stem Cell Transplantation Unit, Antalya and Göztepe Medicalpark Hospitals, Antalya, Turkey; ^30^Department of Pediatric Bone Marrow Transplantation Unit, Medicalpark Göztepe Hospital, Istanbul, Turkey; ^31^Department of Bone Marrow Transplantation, Great Ormond Street Hospital for Children, National Health Service Foundation Trust, London, United Kingdom; ^32^Division of Molecular Hematology, Lund University, Lund, Sweden; ^33^Department of Pediatrics, Oulu University Hospital, Oulu, Finland; ^34^Department of Pediatric Oncology, Hematology and Hematopoietic Stem Cell Transplantation, Poznan University of Medical Sciences, Poznan, Poland; ^35^Pediatric Hematology Oncology, Department of Mother and Child, Azienda Ospedaliera Universitaria Integrata, Verona, Italy; ^36^European Society for Blood and Marrow Transplantation Paris Office, Hôpital Saint Antoine, Paris, France; ^37^Department of Pediatric Hematology, Oncology and Stem Cell Transplantation, University Hospital of Regensburg, Regensburg, Germany

**Keywords:** infection precaution, allogeneic hematological stem cell transplantation, children, antibiotic prophylactic therapy, vaccination

## Abstract

Specific protocols define eligibility, conditioning, donor selection, graft composition and prophylaxis of graft vs. host disease for children and young adults undergoing hematopoietic stem cell transplant (HSCT). However, international protocols rarely, if ever, detail supportive care, including pharmaceutical infection prophylaxis, physical protection with face masks and cohort isolation or food restrictions. Supportive care suffers from a lack of scientific evidence and implementation of practices in the transplant centers brings extensive restrictions to the child's and family's daily life after HSCT. Therefore, the Board of the Pediatric Diseases Working Party (PDWP) of the European Society for Blood and Marrow Transplantation (EBMT) held a series of dedicated workshops since 2017 with the aim of initiating the production of a set of minimal recommendations. The present paper describes the consensus reached within the field of infection prophylaxis.

## Introduction

Infectious complications are a major cause of morbidity and mortality in children undergoing hematopoietic stem cell transplantation (HSCT). In the early history of HSCT many patients died due to reactivated viruses of the herpes family and *de novo* infections especially by fungi and respiratory viruses. Modern HSCT programs therefore rely heavily on efficient infection prevention and early treatment of infections to continue the significant improvement in transplant related mortality (TRM) experienced during the past decades.

Measures to avoid exposure to infectious pathogens include amongst others cohort isolation, use of physical barriers, pharmaceutical prophylaxis, food restriction and vaccinations. In order to help the families and health care workers on how to manage infection risk, local supportive care guidelines are issued in most HSCT centers. However, such guidelines are often based on local preferences rather than on evidence-based studies due to difficulties of carrying out controlled studies in this field. Yet, comprehensive international guidelines were published in 2009 for non-pharmacologic infection prevention during HSCT. The guidelines included the use of HEPA filtered rooms, isolation precautions, restriction of certain food items and crowd isolation (1, 2). Recently, such general recommendations were reviewed in the 2019 handbook issued by the European Society for Blood and Marrow Transplantation (EBMT) (3).

In order to provide an updated and comprehensive set of recommendations directed specifically for children and young adults, the Board of the Pediatric Diseases Working Party (PDWP) of the EBMT decided to review the current evidence within infection prevention in children, during the work on supportive care in pediatric HSCT.

The work was based on repeated focused meetings of the board of PDWP and experts within the fields of pediatric HSCT: Different topics on supportive care were each prepared and finalized by a sub-committee within a total of three meetings held between October 2017 and November 2018 as detailed in the initial paper by Nava et al. (4). Briefly, prior to the initial meeting experts from each sub-committee reviewed the literature and presented during the first meeting a list of issues to be addressed and a draft proposal for the structure and major content of a consensus statement. Based on thorough discussions within the sub-committee and then the entire group, the major objectives/topics and most substantial pieces of evidence but also uncertainties were identified and formed the basis for discussing a more detailed consensus recommendation during the second meeting. During the third meeting the final recommendations were agreed upon within each sub-committee and in consensus of all workshop attendants.

The current publication is the 3rd volume following these supportive care workshops and presents the consensus of infection prevention in pediatric HSCT recipients with a special focus on an updated vaccination program and guidelines for restrictions following discharge until full de-isolation. Following re-evaluation with two further virtual meetings in April 2020 and February 2021, the manuscript also includes considerations regarding Coronavirus disease 2019 (COVID-19).

## Protective Measures at the HSCT Ward

In general, it is recommended to reduce all risks of community acquired infections. The level of inpatient isolation varies between centers due to local logistics and priorities but also due to lack of evidence of specific actions. We here provide the updated recommendations based on international guidelines (1, 2, 5–8) and on the practice in the highly specialized pediatric EBMT centers involved in the workshops. Recently, specific guidelines for SARS-CoV-2 prevention and control have been worked out by scientific societies, including the EBMT (9).

### Rooms

Allo-HSCT recipients should be treated in a highly shielded environment with single patient rooms, preferentially with HEPA filters for prevention of airborne fungal infections, especially *Aspergillus* (1). Filters should be replaced regularly according to the manufacturer's recommendations, especially in centers with ongoing construction work (10, 11). There should be at least 12 air exchanges per hour, keeping a consistent positive air pressure of at least 2.5 Pa between the patient room and hallway (1, 12). In case of a recipient affected with disease transmissible by droplets (i.e., COVID-19, measles), a switch to negative pressure might be considered, in order to protect the ward.

### Barrier Precautions

When entering and leaving the room and before and after patient contact, hand hygiene including alcohol-based hand rubs/hand washing with soap (plain or antimicrobial) and water is absolutely essential (13, 14). In the absence of visible soiling of hands or potential contact with spore-forming organisms (e.g., *Clostridium difficile*), alcohol-based hand rub can be used. Rings, bracelets, artificial nails and adhesive bandages should be avoided. Personal protective equipment (gloves, masks, and gowns) should be worn during procedures generating splashes of body fluids or causing soiling of clothes. Additionally, when indicated on the basis of coexisting conditions, patients should be placed on airborne, droplet or contact precautions (7). Toys including games, videos, mobile phones, and tablet computers should be wiped with certified cleansing material before being brought into the room and thereafter at least once weekly (15–17). Plants, dried or fresh flowers are prohibited. Each patient should have designated examination tools and routine examination equipment should not be transferred from room to room.

### Health Care Workers and Visitors

All HCWs should be vaccinated according to national guidelines including annual influenza vaccination (18). A HCW with known or suspected transmissible infection should have no direct contact with the patients or other HCW and if possible, should be temporarily referred to back-office tasks, or in the case of COVID19 pandemic stay home according to guidelines (19). Visitors should be restricted to as few as possible and any showing signs of infection should be excluded from the transplant unit and from direct contact with HSCT recipients or candidates undergoing conditioning therapy until all symptoms have resolved (1). No absolute minimum age for visitors can be recommended, some centers accept visitors older than 12 years old including siblings, whereas other centers accept even younger visitors. All visitors, including children must be able to follow strict hand hygiene and isolation precautions. Furthermore, during the COVID-19 pandemic, strict adherence to masks by staff and visitors must be followed. Visitor restrictions to transplant units often imply one parent only following negative SARS-CoV-2 testing. The visit of the second parent is rarely allowed, except in case of end-of-life situations.

Given the suboptimal immunogenicity in HSCT patients, family members and healthcare professionals involved in the care of these populations should be vaccinated during influenza seasons, prior to and at least 12 months after HSCT, till patients are able to get and respond efficiently to vaccination. Furthermore, measles and chicken pox status of the family should be reviewed.

## Dietary Restriction During Admission

Enteral nutrition is generally encouraged in order to preserve the natural microbiota, thus reducing the risk of graft vs. host disease (GvHD) and possibly the speed of platelet recovery (20). Conditioning-associated nausea and mucositis generally leads to a reduction in the appetite and oral food intake during HSCT, especially during the neutropenic phase (4). Traditionally, certain dietary items have been widely restricted to reduce the risk of introducing harmful food-borne microorganisms to the HSCT patient (“neutropenic diet”), which is increasingly questioned. A retrospective study analyzed infectious complications in 726 consecutive adult HSCT recipients, 25% receiving an allogeneic transplant (21). A neutropenic diet was provided to 363 patients who underwent HSCT between 2004 and 2006, whereas 363 patients undergoing HSCT after 2006 did not receive a neutropenic diet. The only significant difference between the groups was a higher number of microbiologically documented infections after resolution of neutropenia in patients receiving neutropenic diet. A small prospective and randomized pilot study compared a total of 46 adult patients receiving neutropenic diet or a diet without restrictions from day 1 of conditioning until engraftment and did not find differences in infections (22), which corroborates the results of a meta-analysis including 1,116 patients, with 772 (69.1%) having undergone HSCT (23). Unfortunately, data of the effect of a neutropenic diet in pediatric HSCT is lacking to date, but, superiority of a strict neutropenic diet was not demonstrated in either adults or pediatric oncology patients receiving chemotherapy (24–27).

There is considerable variation in practices in dietary restrictions of specific food items between HSCT-centers (28, 29). However, the growing body of evidence of the lack of benefit of a neutropenic diet has led to an increasing number of cancer centers replacing the strict neutropenic diet with safe food handling guidelines (30, 31). In this regard, the US Department of Agriculture/FDA continues to recommend the avoidance of uncooked ground meat or unpasteurized milk and milk products, whilst categorizing other foods such as fresh vegetables and salad as lower risk food permitted for cancer patients provided certain conditions of food handling and preparation are strictly adhered to (Table 1). Four essential steps (“clean, separate, cook and chill”) are highlighted, and detailed recommendations regarding washing hands and surfaces (“clean”), how to prevent cross-contamination from one food product to another (“separate”), how to cook different food items to safe temperatures (“cook”) and how to refrigerate properly (“chill”) are given.

**Table 1:** Handling of food items during allogeneic hematopoietic cell transplantation.

**Steps**	**Handling and preparing food items**	**Selecting the lower risk option of food items**	
		**Low risk**	**High risk**
Clean	Wash hands and surfaces often. Rinse fruits and vegetables, and rub firm-skin fruits and vegetables under running tap water.	Washed fresh vegetables including lettuce/salads; cooked vegetables	Unwashed fresh vegetables including lettuce/salads
Separate	Separate raw meat, poultry, seafood, and eggs from other foods to avoid cross-contamination (e.g., in the refrigerator, using different cutting boards for raw foods and ready-to-eat food).		
Cook	Cook to safe temperatures, consider using a food thermometer to measure the internal temperature (e.g., beef, lamb, pork, veal and fish to at least 63°C, ground meat to at least 70°C, eggs until yolks and whites are firm). Reheat hot dogs and luncheon meats until steaming hot or 75°C.	Sufficiently cooked meat, poultry, seafood and eggs; canned fish and seafood; pasteurized milk, milk products, egg and egg products	Raw or undercooked meat, poultry, seafood; unpasteurized (raw) milk and milk products Hot dogs and luncheon meats that have not been reheated
Chill	Refrigerate promptly and follow cold storage charts for refrigerator (below 4°C) and freezer (<-16°C). Never thaw food at room temperature.		

In conclusion, replacing the strict neutropenic diet in HSCT recipients with a more palatable diet should not result in an increased risk of infection and would improve the quality of life and further result in an increase in oral intake of calories and protein, helping to prevent undesirable weight loss. However, this consensus statement of European pediatric HSCT-centers needs to be adapted by each center according to regional and local circumstances.

## Pharmaceutical Microbial Prophylaxis During and After HSCT

Exposure to infectious pathogens is unavoidable and necessitates antimicrobial prophylaxis during and after HSCT in all patients. The duration of prophylaxis may depend more on the degree of immune reconstitution in any individual patient rather than any specific time from HSCT. Current evidence has been reviewed and the PDWPs recommendations for the antimicrobial prophylaxis are summarized in Table 2. Local circumstances may modify these recommendations.

**Table 2:** Antimicrobial prophylaxis in children undergoing allogeneic hematopoietic cell transplantation.

**Phase**	**Antibacterial**	**Antiviral**	**Pnemocystis/toxoplasmosis**	**Antifungal**	**IVIG**
Conditioning	NGR	NGR	NGR	NGR	NGR
Pre-engraftment	NGR	aciclovir	NGR	L-AmB, azoles or echinocandins	NGR
Post-engraftment w/o a/c GvHD	NGR	aciclovir	SMX/TMP	NGR	NGR
Post-engraftment with a/c GvHD	PNC	aciclovir	SMX/TMP	azoles or echinocandins	NGR

*NGR, not generally recommended; PNC, penicillin; SMX/TMP, sulfamethoxazole/trimethoprim; L-AmB, intravenous liposomal amphotericin B; IVIG, intravenous immunoglobulin*.

*If IgG levels below 400 mg/dL IVIG could be given. a/c GvHD, acute or chronic graft vs host disease*.

### Pre-assessment

Thorough pre-HSCT assessment including virus specific antibody titers, syphilis and toxoplasmosis is a pre-requisite for the risk management of either reactivation or *de novo* infection and can guide prophylactic treatment pre- and post-discharge. Patients testing IgG-seronegative should in general remain on prophylactic treatment to avoid *de novo* infection prior to HSCT. Interferon-gamma-release assay (IGRA) rather than a tuberculin skin test, is recommended if tuberculosis is clinically suspected. Occasionally in non-acute HSCT, the time from initial referral of the child until HSCT may allow for considering targeted antimicrobial therapy or (re)- immunization and/or immunoglobulin prior to transplant. Pre-transplant serology may not be relevant for anamnestic IgG in those who were recently exposed to products containing plasma (fresh frozen plasma, platelet concentrates, granulocyte transfusions, …) or received intravenous immunoglobulin infusion.

### Antibacterial Prophylaxis

Systemic antibacterial prophylaxis and selective gut decontamination is no longer recommended during the neutropenic period (32–35). Instead, immediate administration of intravenous antibiotic treatment when infection is suspected is mandatory. Empiric antibiotic treatment should be adapted to local resistance patterns, patients' colonization status and cover gram-negative aerobic bacteria (*Enterobacteriaceae* and Pseudomonas aeruginosa) and gram-positive cocci (streptococci, Staphylococcus aureus and *Enterococci*). A strict implementation of the guidelines for treatment of febrile neutropenia is mandatory (35–37).

Late infection prevention (>100 days posttransplant), targeting mainly encapsulated bacteria (*Streptococcus pneumoniae* and *Haemophilus influenzae*), should include penicillin or macrolide antibiotics during immunosuppressive treatment for GvHD (38, 39), immunoglobulin replacement therapy (i.v. or s.c.) in patients with severe hypogammaglobulinemia (serum IgG level <400 mg/dL) and vaccinations (see later).

### Antiviral Prophylaxis

The risk of HSV disease after allo-HSCT in the absence of prophylaxis is ~80%, especially during the first neutropenic period after HSCT or associated with stomatitis. Prophylaxis with aciclovir (ACV), at a standard dose of 2 × 20 mg/kg from day +1 is recommended in all HSV seropositive patients until neutrophil engraftment or mucosal recovery (3, 40). However, the emergence of drug resistance to ACV may hamper the efficacy of HSV prophylaxis, thus ACV-resistant HSV isolates has been reported in up to 30% among allogeneic bone marrow transplant patients (41).

Following discharge, it is recommended that VZV-seropositive patients receive ACV (2 × 20 mg/kg or 2 × 800 mg in adolescents) or valaciclovir (2 × 500 mg in adolescents) until day +365. There may be an increased risk of delayed VZV-reactivation after discontinuing ACV (rebound) and optimal ACV dose and duration of the prophylaxis is still to be defined (40, 42). Post-exposure prophylaxis with anti-VZV-immunoglobulins (within 96 h) and ACV/valaciclovir is recommended for seronegative patients exposed to VZV. Prophylaxis should be started as soon as possible and continued until 21 days after exposure (43). Primary VZV-infection after allo-HSCT is associated with a high mortality and should be avoided by isolation measures or vaccination (2). Cytomegalovirus (CMV) is a well-known cause of disease occurring after allogeneic HSCT. The manifestations of CMV range from asymptomatic infection, defined as active CMV replication in the blood in the absence of clinical and organ manifestations to CMV disease, characterized by CMV infection with clinical symptoms and/or organ function abnormalities. Fever, cytopenia, pneumonia, gastrointestinal involvement and retinitis are the most frequent presentations. CMV prophylaxis or pre-emptive therapy has changed the natural history of the disease, reducing the risk of CMV disease, CMV-associated death and transplant-related mortality.

Prevention of CMV disease is a prerequisite in allo-HSCT and includes preference of CMV-matched donor/recipient pairs and prevention of virus exposure by discontinuation of breast feeding by CMV-positive mothers (44), physical protection measures (isolation) and use of leukodepleted and/or gamma-irradiated blood products. A preemptive strategy based on weekly monitoring of CMV replication is strongly recommended using foscarnet before and ganciclovir after stable neutrophil engraftment in the presence of rising CMV-loads (45). CMV-monitoring should continue until acceptable immune recovery and/or tapering of immunosuppressive therapy (IST). CMV prophylaxis with letermovir is recommended in adult CMV-positive HSCT recipients (45) and may be an option for seropositive children in an off-label setting (46).

Prevention of EBV-disease following HSCT relies on prevention of exposure (isolation) and preemptive strategies in EBV-seropositive patients (47, 48). Post-transplant lymphoproliferative disease (PTLD) is the most frequent EBV-associated transplant related disease. For timely diagnosis, it is recommended to weekly monitor by quantitative PCR until immune recovery. In case of rapidly increasing viral loads, preemptive treatment with Rituximab is recommended (2, 49). Rituximab-induced B cell depletion has a prophylactic role in controlling EBV-DNAemia after HSCT. Rituximab targets CD20+ B cells which proliferate under the viral trigger, thus removing the EBV reservoir. However, it is not clear if rituximab improves survival, beyond reducing EBV DNAemia. Therefore, the latest ECIL-6 guidelines only recommend prophylactic rituximab for patients at very high risk of developing PTLD, with surveillance of hypogammaglobulinemia and prompt administration of intravenous immunoglobulins to limit rituximab-related complications (12). EBV-specific cytotoxic T lymphocytes (CTLs) showed promising results but remain limited to second-line settings. Antiviral agents are ineffective *in vivo* since their target is not expressed by latent B cells (50).

Primary infections or reactivations of other respiratory tract viruses (RSV, parainfluenza, influenza, and adenovirus) are associated with severe illness and should be monitored closely. Prevention of disease relies on preventing exposure before and after HSCT, see below. HHV-6, parvovirus B19 and the polyomaviruses (BK and JC) are associated with specific clinical entities in allo-HSCT recipients. However, no established prophylaxis exists for these and no recommendations can be made currently (51).

### Antifungal Prophylaxis

Invasive fungal infections (IFI) remain a challenge due to substantial morbidity and mortality, despite the availability of new antifungal drugs such as broad spectrum triazoles or echinocandins. Primary antifungal prophylaxis is strongly recommended irrespective of primary diagnosis during the neutropenic phase and until immune reconstitution and in situations with augmented immunosuppression due to GvHD, which is in line with the updated guidelines according to the Eighth Conference on Infections in Leukemia (ECIL-8) (35). We recommend practice according to the ECIL-8 guidelines to include fluconazole (only if the institutional incidence of invasive mold infections is low) or voriconazole (therapeutic drug monitoring (TDM) strongly recommended), posaconazole (not approved for children, TDM recommended); itraconazole (not approved for children), liposomal amphotericin B (off-label) or micafungin. Other options against pulmonary mold infection may include aerosolized liposomal amphotericin B or caspofungin (off-label indication). Secondary prophylaxis is highly recommended in patients with IFI prior to allo-HSCT.

Monitoring of *Aspergillus* using serum galactomannan levels is feasible, yet the negative predictive value is relatively high and other molds remain undetected (37). According to ECIL-8 guidelines galactomannan monitoring is valuable in children not receiving mold-active prophylaxis, but experts discourage its value in those receiving mold-active prophylaxis (35).

The choice of antifungal drug during and after HSCT should be determined by local circumstances and resistance patterns, organ toxicity (i.e., nephrotoxicity) and drug-drug interactions (e.g., azoles with calcineurin inhibitors) (52). During conditioning, azoles are not recommended whereas L-Amb (off-label) may be preferred until discharge, though nephrotoxicity may limit its use. Triazoles incl. posaconazole have numerous drug interactions including interaction with calcineurin inhibitors that must be considered, and therapeutic drug monitoring is mandatory (53). At the time of writing, posaconazole is still not licensed for recipients <18 years in the EU.

### Prophylaxis Against Toxoplasmosis

Toxoplasmosis is a widely distributed zoonosis produced by the parasite *T. gondii* and continues to be a major challenge in the management of pediatric allogeneic HSCT recipients (54). The incidence of toxoplasmosis varies widely due to variable seroprevalence among patient populations, but a majority of patients (up to 3/4) with post HSCT toxoplasmosis disease were IgG-positive prior to HSCT, thus indicating that reactivation plays a major role rather than *de novo* infection (55, 56). Most cases of *Toxoplasma* are observed between 2 and 6 months after transplant with a risk persisting across the first year of transplant and sometimes even later. Early cases occurring during the first 4 weeks of transplant are very rare but have a high mortality rate. Prevention of disease by pre-HSCT assessment of sero-positivity and either prophylaxis with trimethoprim/sulfamethoxazole or preemptive strategy with weekly PCR-monitoring is recommended in seropositive patients starting immediately after transplant until 6 months post HSCT (2, 56).

### Prophylaxis Against Pneumocystis Pneumonia

Prior to routine *Pneumocystis jirovecii* pneumonia (PJP) prophylaxis, the cumulative incidence of PJP after allo-HSCT was estimated at 9–16%. Early mortality rates are high, and prophylaxis is strongly recommended after engraftment until at least day +180. Trimethoprim/sulfamethoxazole given 2–3 times weekly is the drug of choice for the primary prophylaxis of PJP in adults and children and should be given during the entire period at risk. All other drugs, including pentamidine, atovaquone and dapsone, are considered second-line alternatives when trimethoprim/sulfamethoxazole is poorly tolerated or contraindicated and are preferred over inhaled pentamidine (57). PJP prophylaxis is relevant in patients with delayed immune reconstitution (CD4 T-cell <200/μL) or patients receiving IST for GvHD (58).

### Prophylaxis Against COVID-19

The Coronavirus disease 2019 (COVID-19), caused by the severe acute respiratory syndrome Coronavirus 2 (SARS-CoV-2), continues to expand worldwide, since it was declared a pandemic by the WHO in March 2020 (59). One year later, 116 million confirmed cases and 2.6 million deaths worldwide have been reported to WHO [World Health Organization Coronavirus (COVID-19) Dashboard https://covid19.who.int]. A cancer diagnosis in adults doubles the risk of COVID-19-associated intensive care unit admission and death, compared with the general population (60, 61). Data regarding COVID-19 in transplanted pediatric patients are very scarce. Despite pediatric cancer patients mostly presenting with a mild or asymptomatic course, the risk of severe COVID-19 may be higher compared with the general pediatric population (62, 63). The Center for International Blood and Marrow Transplant Research (CIBMTR) recently reported a 68% survival 1 month after COVID-19 diagnosis in 318 transplant recipients of whom only 29 of were children, adolescents or young adults. The mortality risk was 2.7 higher in case the infection occurred in the first 12 months after transplant (61). Organ toxicities induced by chemo/radiotherapy, especially in the lungs, and risks of additional infections, due to pancytopenia and immunosuppression, are likely to contribute to exacerbate the COVID-19 course, even though an ultimately protective role of immunosuppression cannot be ruled out (61).

National and local guidelines, policies and procedures should be followed, as the COVID-19 situation varies amongst regions. Yet, the EBMT provides guidelines on COVID precautions in the EBMT-setting, including considerations in pediatric HSCT (9). These guidelines are continuously updated on the EBMT website (https://www.ebmt.org/sites/default/files/2021-02/EBMT%20COVID-19%20guidelines%20v.%2015.02%202021-02-18.pdf). The section dealing with prevention policies confirms case tracking and isolation of SARS-CoV-2 infected individuals, hand hygiene, masks and social distancing as the main prevention strategy. Furthermore, patients and donors should be tested upon admission and/or before the conditioning regimen starts. Visits in the outpatient setting should be limited to patients who are in real need, whereas long-term patients may be followed by telemedicine. The risks associated with postponing scheduled appointments should be balanced (59).

Transplant deferral during the pandemic may be considered in non-urgent patients, as hemoglobinopathies, even though a careful evaluation of the risk-benefit balance is recommended.

In case of recipient SARS-CoV-2 positivity, transplantation should be delayed up to two consecutive negative results, even though persistence of virus positivity for several weeks or months in severely immunocompromised patients is not uncommonly reported (61). Donation from a SARS-CoV-2 positive donor should also be deferred. Graft cryopreservation is recommended during the peaks of the pandemic, due to travel restrictions which may prolong its delivery time. The impact of cryopreservation on primary and long-term engraftment is still to be assessed, but many centers prefer mobilized peripheral blood stem cell to ensure good counts, also after thawing, in order not to jeopardize engraftment (60).

## Post Discharge Precautions

On discharge after allo-HSCT, most patients are still severely immune compromised. Local logistical and geographical factors determine whether patients are discharged to their home or to a local patient hotel or alike. In either case, individual precautions to avoid community acquired infections during immune reconstitution, must be taken. The considerable risk of morbidity or mortality due to acquired infection should be minimized by avoiding microbial exposure. However, no studies support specific approaches of how to protect the child or young adult from community acquired infections. Instead, a number of observations have historically led to recommendations regarding social distancing, food intake and exposure to indoor or outdoor microbes (1, 7, 64–66). The scope of the precautions is to reduce the risk of exposure to especially RSV, *Influenza A, B and C, Parainfluenza, Coronavirus, Norovirus, Rota virus, Adenovirus, Salmonella Enteritidis, Toxoplasma and Aspergillus* species and should be comprehensive in children due to high rates of airborne infections amongst other children below 6–8 years.

As a part of the consensus workshops, a survey demonstrated that most centers recommend avoiding crowds during the first year post HSCT by not attending school or kindergarten and by avoiding public transportation etc., but the timing of return to normal activities differs widely.

Therefore, in order to provide expert based guidelines on these complex scenarios, we developed a schematic algorithm during the three workshops recommending a set of minimal precautions that should be implemented based on risk stratification of the individual transplanted child. During the workshops we constructed the algorithm defined by three different risk groups, defined as low, intermediate or high risk. The risk grouping is based on immune recovery (CD4 positive T-cells and granulocytes), presence of GvHD, level of IST and infection rate (recurrent viral re-activation, recurrent airway infections), see Table 3. According to this, low risk patients are GvHD-free, off IST, do not experience recurrent infections and CD4 counts are >200/μL. High risk group patients have uncontrolled GvHD, receive multimodal IST, have viral reactivations or CD4 counts are <100/μL, whereas intermediate risk group are in between. Patients may move between risk groups in the event of occurrence/recurrence of GvHD or frequent infections.

**Table 3:** Risk grouping following discharge from allogeneic hematopoietic cell transplantation in childhood or adolescence.

**Risk assessment at time of discharge, day 100, day 180**		
**Low risk**	**Int risk**	**High risk**
No GvHD	No or controlled GvHD	Active/uncontrolled GvHD
No IST	Low IST	Multimodal IST
No infections	Recurrent infections	Viral reactivations
CD4 > 200	CD4 100-200	CD4 <100
*N* > 0.5 × 10^9^/l	*N* ≤ 0.5 × 10^9^/l	*N* ≤ 0.5 × 10^9^/l

*GvHD, graft vs. host disease; IST, immune suppressive therapy; CD4, CD positive T-lymphocytes; N, neutrophil count*.

**Figure 1:**
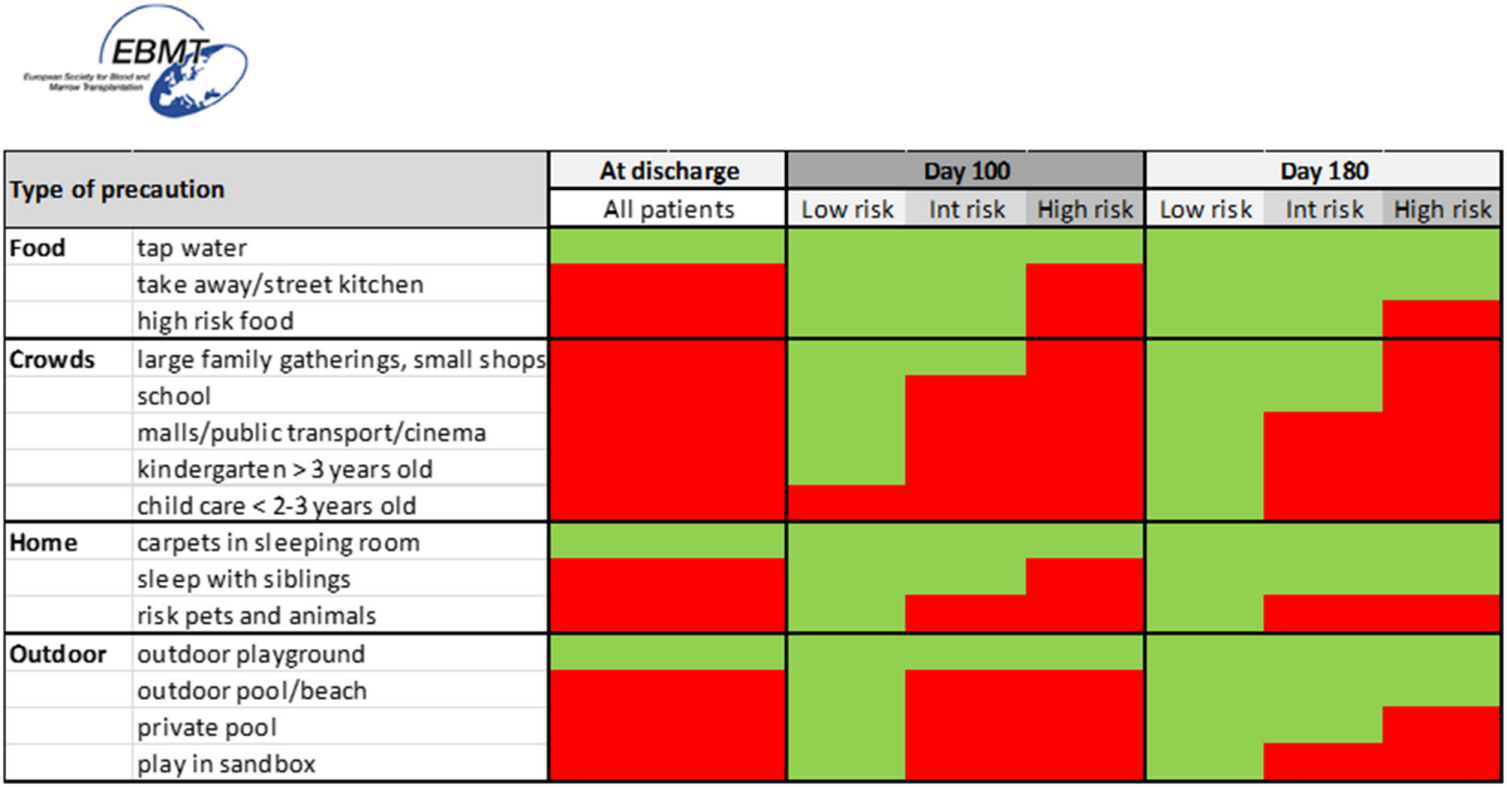
Schematic algorithm for protective measures following discharge from allogeneic hematopoietic cell transplantation in childhood or adolescence. Red color indicates not recommended, green color indicates no restrictions. High risk foods: unpasteurized milk; undercooked egg; blue cheese; honey; raw and undercooked fish, meat and seafood; selected nuts; dried spices from uncontrolled shops/markets; unwashed or unpeeled fresh fruit, water from private wells. Nursery: 0–3 years. Kindergarten: 3–5/6 years. High risk pets: turtles (yersinia), shifting cat trays (toxoplasma), visit stables (aspergillus), acquire new pets. Private pool: if not at bacterial infection. Tap water: local outbreaks may moderate.

This risk stratification can then be used to guide patients and their families to a risk adapted set of precautions listed in Figure 1. We suggest that such risk classification is done at three different timepoints post HSCT: timepoint 1 at initial discharge post-HSCT, timepoint 2 approximately day +100 and timepoint 3 at approximately day +180. At each timepoints, a patient should be stratified according to the risk group (Table 3), which then indicate the set of precautions that should be kept.

The precautionary measures agreed upon by the experts during the workshops include food restrictions, social distancing and behavior at home and outdoors. Importantly though, local (geographical) specific circumstances and outbreaks in infection should be considered and food restrictions may be adjusted accordingly. In principle, the more people in a crowd the higher the risk for transfer of airborne microbes. Thus, childcare centers, indoor waterparks, malls, cinemas and public transportation are regarded as high risk, whereas small family gatherings or small restaurants are regarded intermediate risk. Individual circumstances should be considered when relaxing restrictions as a return to a more normal life may improve the general well-being of the child.

Specific counseling of the family could according to the work shops include:

If possible, patients should not return to a home with high mold and re-housing should be considered until the problem is fixed. Sharing the bedroom with sick siblings should be avoided if possible. Furthermore, the acquisition of new pets and shifting a cat litter box should be avoided in the early post HSCT period.

Outdoor precautions include avoiding bathing in pools and playing in sandboxes. Bathing in the ocean is considered safe, provided the patient has no indwelling catheters.

## Vaccinations

**Figure 2:**
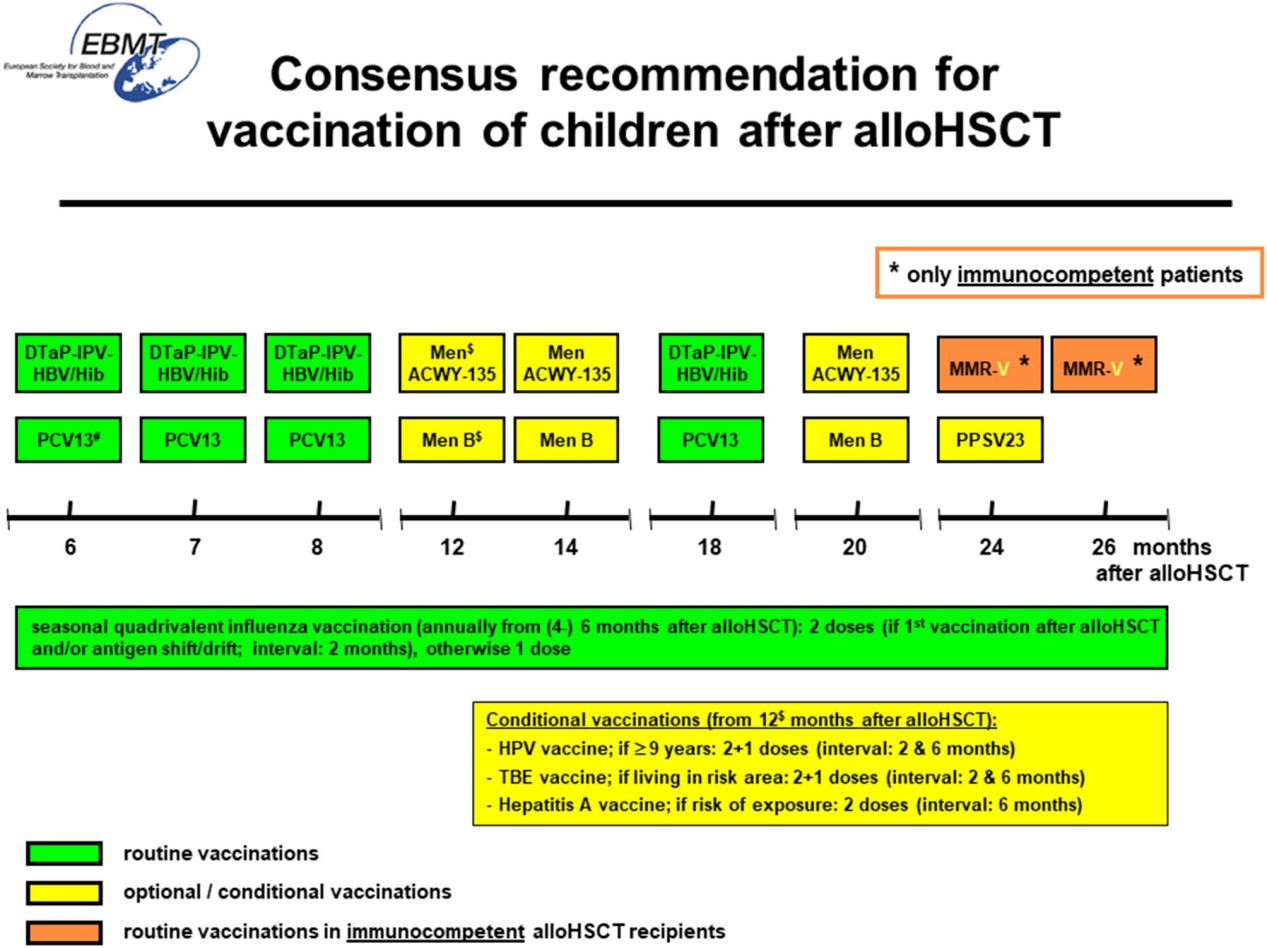
Recommended and optional/conditional vaccinations after allogeneic hematopoietic cell transplantation in childhood. DTaP-IPV-HBV/Hib, hexavalent diphtheria, tetanus, acellular pertussis, inactivated poliovirus, hepatitis B, *Haemophilus influenzae type B* vaccine; PCV13, 13-valent pneumococcal conjugate vaccine; Men ACWY-135, quadrivalent meningococcal conjugate vaccine; Men B, recombinant meningococcal type B vaccine; PPSV23, 23-valent pneumococcal polysaccharide vaccine; MMR-V, live attenuated measles, mumps, rubella, varicella-zoster-virus vaccine; HPV, human-papilloma-virus vaccine; TBE, tick-born-encephalitis vaccine; HAV, hepatitis A vaccine. ^#^ start of vaccination at 3 months post-HSCT possible after individual risk-benefit analysis (please refer to text).^$^ start of vaccination at 6 months post-HCT possible after individual risk-benefit analysis (please refer to text). * only immunocompetent patients post-HSCT, if ≥ 3 months without immunosuppressive therapy and ≥ 3 months without active cGvHD.

Children undergoing HSCT lose immunity to vaccine-preventable diseases and are exposed to various pathogens upon reintegration into social life with potentially life-threatening pneumococcal infections playing a prominent role. Thus, early and comprehensive re-immunization is important post-HSCT and should recognize stepwise recovery of the immune system after HSCT and potential impact of IST for prophylaxis/treatment of GvHD that impacts immune recovery. Considerations on the timing of re-vaccination post-HSCT must balance the clinical demand for swift protection and the risk of immunization failure if vaccination is given too early. In the setting of this background an expert group of pediatric infectious disease and transplant physicians identified the specific clinical demand and reviewed currently available evidence focussing on the pediatric age group and generated the consensus recommendation detailed in Figure 2.

### General Principles for Immunization Post-HSCT in Children

Based on data from a retrospective analysis of revaccination of pediatric HSCT recipients from Great-Britain (67), the prospective IKAST trial on vaccination of children after HSCT (68) and a trial on 13-valent pneumococcal conjugate vaccination in HSCT recipients (69) the following recommendations are made (Table 4):

– Use a fixed starting time point for re-vaccination with the newborn DTaP/IPV/HBV/Hib combination vaccine and the 13-valent pneumococcal conjugate (PCV13) vaccine six months post-HSCT [if leukocyte engraftment and platelets ≥ 50 × 10(9)/l] and immunize irrespective of donor/graft type, GvHD, IST and/or measures of immune recovery.
– Use combination vaccine DTaP/IPV/HBV/Hib irrespective of chronologic age.
– Optional/conditional vaccinations (highlighted in yellow in Figure 2) should not interfere with evidence-based immunizations (DTaP/IPV/HBV/Hib, PCV13) starting at 6 months. Optional/conditional vaccinations preferably start 12 months post-HSCT.
– Immunization with non-live vaccines is safe during IVIG replacement as there is no specific risk besides non-response. Check titres 3 months after stopping IVIG.
– Start vaccination with live vaccines (MMR-V) not earlier than 24 months post-HSCT and restrict to immunocompetent patients without GvHD and IST ≥ 3 months and off IVIG substitution.
– Consider checking antibody concentrations prior and 1 month after primary series in patients with GvHD, IST, IVIG treatment and/or delayed immune reconstitution.

**Table 4:** Recommendations on specific vaccines following allogeneic hematopoietic cell transplantation in childhood.

**Vaccine**	Start^*^	**No of doses**	Schedule^**^	**Specific notes**
**Routine vaccinations**				
Hexavalent diphtheria, tetanus, acellular pertussis, inactivated poliovirus, Hepatitis B, *Haemophilus influenzae type B* vaccine (DTaP-IPV-HBV/*Hib*)	6	4	0-1-2-12	
13-valent pneumococcal conjugate vaccine (PCV13)	6	4	0-1-2-12	PCV13 comprises most serotypes in invasive pneumococcal disease post-HSCT
Quadrivalent, inactivated influenza virus vaccine (comprehensive current seasonal strain coverage)	(4-)6	1-2	0-2	For first vaccination post-HSCT or after substantial antigenic shift/drift two doses should be given. Yearly revaccination is recommended. Early start at 4 months post-HSCT is possible in case of pandemia or up-coming influenza season. Live influenza vaccine is contra-indicated in HSCT recipients.
Live, attenuated measles- mumps-rubella virus vaccine (MMR)	24	2	0-2	*If immunocompetent (≥ 3 months without GvHD/immunosuppression)*
**Conditional vaccinations**				
Human papilloma virus vaccine (HPV)	12	3	0-2-8	Substantial risk for HPV-related cancer post-HSCT. Vaccination of both boys and girls *if age ≥ 9years*
Hepatitis A vaccine (HAV)	12	2	0-6	*If risk of exposure*
Tick-borne encephalitis vaccine (TBE)	12	3	0-2-8	*If living in endemic region*
**Optional vaccinations**				
Live, attenuated varicella-zoster-virus vaccine (in combination with MMR; MMR-V)	24	2	0−2	*If immunocompetent (≥ 3 months without GvHD or immunosuppression*. VZV-reactivations frequently occur before 24 months and are thus beyond effect of the live vaccine which can only be administered from 24 months post-HSCT.
Quadrivalent meningococcal type A,C,W,Y-135 conjugate vaccine (Men ACWY-135)	12	3	0-2-8	No data on specific risk of invasive meningococcal disease post-HSCT. Few and disappointing data for tetra-valent conjugate vaccine post-HSCT.
Recombinant meningococcal type B vaccine (Men B)	12	3	0-2-8	No reported experience with MenB vaccination after HSCT.
23-valent pneumococcal polysaccharide vaccine (PPSV23)	24	1	n/a	PPSV23 may broaden serotype protection. Sparse data on the immunogenicity of PPSV23 with regard to the serotypes beyond PCV13.

* *Months from HSCT*.

** *Months from start of vaccination*.

Additional note: Single-center experience indicates that providing non-live vaccines earlier than 6 months post-HSCT may be feasible in children with very swift immune recovery. However, there are no published data on this policy and limited induction of immunologic memory and duration of protection must carefully be weighed against potential earlier protection.

### Specific Recommendation for Selected Vaccines

#### Influenza

High risk for life-threatening influenza-virus infection post-HSCT mandates annual immunization with inactivated influenza vaccines comprising quadrivalent strain coverage. Two doses should be given for first influenza vaccination post-HSCT and after antigenic shift/drift. Live influenza vaccine is contra-indicated post-HSCT. Starting influenza vaccination at 4 months after transplantation may be considered in cases of influenza pandemic or upcoming season.

#### Pneumococcus

PCV13 comprises the majority of pneumococcal serotypes detected in invasive pneumococcal disease post-HSCT. Administration of the 23-valent pneumococcal polysaccharide vaccine (PPSV23) at 24 months may broaden protection. However, sparse data on the immunogenicity of PPV23 with regard to serotypes reaching beyond PCV13 result in an optional recommendation for PPSV23 post-HSCT.

#### Meningococcus

No data exist on the specific risk for invasive meningococcal disease post-HSCT but immunecompromised patients represent candidates for meningococcal vaccination. Clinical relevant protection requires vaccination with both A/C/W/Y135 conjugate and recombinant MenB vaccines. Only few disappointing data are available for A/C/W/Y135 conjugate and no data with MenB vaccination post-HSCT resulting in an optional recommendation for meningococcal vaccination starting at 12 months post-HSCT.

#### Human Papilloma Virus

Profound risk of HPV-associated squamous cell carcinoma exists post-HSCT and most European countries recommend universal HPV vaccination. Consequently, all adolescent transplant recipients should receive HPV vaccination starting from 12 months post-HSCT.

#### Varicella-Zoster-Virus

High incidence of VZV reactivations with substantial morbidity exists in the first 2 years post-HSCT. Only few case-series report on the use of the live-attenuated VZV vaccine in pediatric HSCT recipients. Immunization can only be instituted at 2 years after HSCT coming too late to prevent the major burden of VZV reactivation. These considerations lead to an optional recommendation for immunization with live-attenuated VZV vaccine in immunocompetent children at least 24 months post-HSCT. Vaccination of family members and household contacts is urgently recommended. If a post-vaccination rash develops the vaccinated should avoid contact with HSCT recipients who may receive aciclovir prophylaxis. Non-live VZV vaccines have recently been investigated in immunocompromised hosts. An inactivated VZV vaccine as well as an adjuvanted VZV subunit vaccine prevented zoster reactivation in adult autologous HSCT recipients. No data are available for either of these vaccines in the post-HSCT setting. Thus, no recommendation can be made for their use in children post-HSCT.

#### COVID-19

During the COVID-19 pandemic, it seems prudent to administer COVID-19 vaccines—under the condition that they are non-live and non-replicating—to all recipients of allogeneic HSCT, as soon and as far as they are available for use in children and adolescents (of note: the Pfizer/BioNTec COVID-19 vaccine label includes adolescents 12 years or older). Nevertheless, clinical trials are going on to assess safety and efficacy also in younger patients. In analogy with the recommendation for the influenza vaccination, in the current active pandemic situation, the immunization may be started earlier than 6 months after alloHSCT e.g., at 3 months - but it is recommended to do antibody assessments whenever available prior to and 4 weeks after (last) vaccination of the primary series in order to assess immunogenicity as information on this is lacking, in particular in the setting of pediatric alloSCT. This recommendation is in accordance with the current EBMT guideline for COVID-19 vaccination in allogeneic HSCT recipients (https://www.ebmt.org/sites/default/files/2021-02/COVID%20vaccine%20version%204.03%20with%20table.pdf).

More extensive data on the rationale and risk-benefit assessment for specific vaccines can be found in two international consensus documents on vaccination in HSCT recipients (70, 71).

## Conclusive Remarks

The emergence and rapid worldwide spread of SARS-COV-2 during early 2020 had substantial impact on general health care and for children and adolescents with the need for allogeneic HSCT it exposed the necessity of strict infection prevention measures. Restrictions regarding hospital resources, donor availability, issues with logistics of stem cell product transport as well as potential consequences of COVID-19 exposure of donors and patients are among the long list of factors potentially impacting provision of care. Nevertheless, the reported incidence of COVID-19 cases among pediatric HSCT recipients is low thus far, which may at least partially reflect the comprehensive measures for social distancing which are implemented as routine standard-of-care in the majority of pediatric HSCT centers. Thus, adherence to the recommendations for preventing infections outlined in this manuscript are considered of particular importance in general but also in the current COVID-19 era.

## Data Availability Statement

The original contributions presented in the study are included in the article/supplementary material, further inquiries can be directed to the corresponding author/s.

## Author Contributions

MI, RM, PS, KK, LS, DH, TL, ABa, and PB wrote the manuscript. PB led the workshops. AJ, TG, JS, IY, HB, MK, MA, TN, J-HD, CD-d-H, ET, UF, MH, MD, MC, CB, ABe, BG, GK, KV, TM, JB, AL, CP, AY, KY, GL, SB, DT, RN, JW, SCe, AD, SCo, and AW participated in workshops and contributed to and reviewed the manuscript. All authors contributed to the article and approved the submitted version.

## Conflict of Interest

The authors declare that the research was conducted in the absence of any commercial or financial relationships that could be construed as a potential conflict of interest.

## Publisher's Note

All claims expressed in this article are solely those of the authors and do not necessarily represent those of their affiliated organizations, or those of the publisher, the editors and the reviewers. Any product that may be evaluated in this article, or claim that may be made by its manufacturer, is not guaranteed or endorsed by the publisher.
